# A randomized clinical trial to evaluate the effects of icodextrin on left ventricular mass index in peritoneal dialysis

**DOI:** 10.1038/s41598-022-20157-z

**Published:** 2022-09-22

**Authors:** Lilian Cordeiro, Walther Yoshiharu Ishikawa, Maria Claudia C. Andreoli, Maria Eugenia F. Canziani, Luiza Karla R. P. Araujo, Benedito J. Pereira, Hugo Abensur, Rosa M. A. Moysés, Rosilene M. Elias

**Affiliations:** 1 Hospital das Clínicas HCFMUSP, Universidade de São Paulo, Rua Dr. Enéas de Carvalho Aguiar 255, 7º andar, São Paulo, SP 05403-000 Brazil; 2 Radiologia, Instituto do Coração do HCFMUSP, Universidade de São Paulo, São Paulo, Brazil; 3 Nefrologia, Universidade Federal de São Paulo (UNIFESP), São Paulo, Brazil; 4 Universidade Nove de Julho (UNINOVE), São Paulo, Brazil

**Keywords:** Cardiac hypertrophy, End-stage renal disease, Peritoneal dialysis

## Abstract

Left ventricular hypertrophy is a risk factor for cardiovascular mortality in patients on peritoneal dialysis (PD). Because icodextrin has a greater ultrafiltration power compared with glucose-based solutions for long dwell, it could improve left ventricular mass by reducing fluid overload. This was a randomized clinical trial that included patients on PD recruited from 2 teaching hospitals, in Sao Paulo—Brazil. Patients were allocated to the control glucose group (GLU) or the intervention icodextrin (ICO) group. Clinical and cardiac magnetic resonance image (MRI) parameters were evaluated at baseline and 6 months after randomization. The primary outcome was the change in left ventricular mass adjusted by surface area (ΔLVMI), measured by cardiac MRI. A total of 22 patients completed the study (GLU, N = 12 and ICO, N = 10). Baseline characteristics such as age, sex, underlying disease, and time on dialysis were similar in both groups. At baseline, 17 patients (77.3%) presented with left ventricular hypertrophy with no difference between groups (p = 0.748). According to the total body water (TBW)/extracellular water (ECW) ratio, 36.8% and 80% of patients from GLU and ICO groups, respectively, were considered hypervolemic (p = 0.044). During follow-up, ΔLVMI was 3.9 g/m (− 10.7, 2.2) in GLU and 5.2 (− 26.8, 16.8) in ICO group (p = 0.651). ΔLVMI correlated with change in brain natriuretic peptide (r = 0.566, p = 0.044), which remained significant in a multiple regression analysis. The use of the icodextrin-based solution in prevalent patients on PD compared with a glucose-based solution was not able to improve LMV. A larger randomized trial with a longer follow-up period may be needed to show changes in LVM in this patient population.

Trial registration: this study has been registered at ReBEC (Registro Brasileiro de Ensaios Clinicos) under the identification #RBR-2mzhmj2, available at: https://ensaiosclinicos.gov.br/pesquisador.

## Introduction

Left ventricular hypertrophy (LVH) is an independent marker for cardiovascular mortality in patients on dialysis^1,2^. Fluid overload is an important contributor to LVH and mortality in this population^3,4^. For patients on peritoneal dialysis (PD), hypervolemia should be targeted considering salt/water intake, residual renal function, and ultrafiltration (UF).

The duration of dwells and solution type should be chosen to optimize peritoneal UF, after considering peritoneal transport characteristics. Icodextrin, a high-molecular-weight glucose polymer, provides superior peritoneal UF compared to glucose-based solutions for long dwell^5^, which is able to remove a higher amount of sodium through small pores^6^. Although icodextrin seems a better therapeutical option to control fluid overload and reduce left ventricular mass in patients on PD, few studies tested this hypothesis^7–12^, and only one applied cardiac magnetic resonance imaging (MRI) to measure ventricular mass^12^. Li et al.^12^ in a multicenter randomized trial compared glucose-based solution vs. icodextrin or other solutions. The authors found no impact on ventricular mass, but the study was focused on metabolic instead of cardiac outcomes.

MRI is more accurate than echocardiography to measure ventricular mass^13^ since it provides lower inter-observer variability and does not overestimate the measurement of left ventricular mass^14,15^. Hence, given the scarcity of data in the literature, the current study applied cardiac MRI to compare the impact of icodextrin vs. glucose-based solution in the changes over time of left ventricular mass in patients on PD.

## Methods

### Study population

Before trial initiation, the study was approved by the Local Ethics Committee Board from both sites (#57602516.6.3001.5505). All included patients have provided a priori written informed consent. Patients ≥ 18 years-old on PD by either continuous ambulatory peritoneal dialysis (CAPD) or continuous cyclic peritoneal dialysis (CCPD) for at least 3 months before randomization were eligible. Exclusion criteria included: American Heart Association Class III/IV congestive heart failure, recent hospitalization or infection (< 30 days), use of pacemakers, and claustrophobia.

### Design and randomization

This was a randomized clinical trial that included patients on PD, recruited from 2 teaching hospitals in Sao Paulo, Brazil, from November 1st, 2016, to August 30th, 2020. Patients were recruited from the Hospital das Clinicas, Universidade de Sao Paulo, and the dialysis unit from the Universidade Federal de Sao Paulo. This research was performed in accordance with the Declaration of Helsinki.

After inclusion and exclusion criteria were checked, subjects were randomized (opaque envelope) to intervention (once a day, long exchange dwell of icodextrin—ICO group) or control (glucose-based solution—GLU group), for at least 6 months. Before study entry, all patients were being treated exclusively with glucose PD fluid. During the study, the prescription of dialysis and medications was at the discretion of the treating nephrologist. The 7.5% icodextrin bag (Extraneal by Baxter) was used during the day in patients on CCPD and overnight in those on CAPD, with a minimal dwell period of 9 h.

### Clinical data retrieval

Clinical and demographic data were obtained in a clinical interview at the study entry and included age, sex, duration of PD, etiology of renal failure, weight, systolic and diastolic blood pressure, presence of diabetes, and average ultrafiltration in the last 30 days, which was obtained from the PD records. Blood pressure was measured using an oscillometric automatic and calibrated monitor (Omron), after resting for at least 5 min. The result obtained from a single measurement was analyzed. In each clinical consultation presence of edema and symptoms such as shortness of breath and dyspnea were evaluated.

Medications were prescribed as determined by the treating physician. We recorded the use of diuretics, phosphate binder, anti-hypertensive drugs, angiotensin-converting enzyme inhibitor/aldosterone receptor blockers (ACE/ARB), cholecalciferol supplementation, calcitriol, and cinacalcet. Changes in these medications were identified and evaluated. Also, diuresis volume was obtained from the latest 24-h laboratory before randomization.

### Laboratory data

Monthly routine laboratory data were recorded. All samples were collected on a weekly basis and processed in the central laboratory from each institution using validated techniques. The following laboratory tests were evaluated: albumin, hemoglobin, hematocrit, urea, creatinine, total calcium, ionized calcium, glucose, glycated hemoglobin, cholesterol, triglycerides, phosphate, alkaline phosphatase, parathyroid hormone (PTH), 25-hydroxyvitamin D, brain natriuretic peptide (BNP), ferritin, C-reactive protein, and 24-h urinary urea for Kt/V calculation. Intact Fibroblast growth factor 23 (FGF-23) was measured using an Elisa assay (Immunotopics, San Clemente CA; RR: 11.7–48.6 pg/ml).

Values of total calcium were corrected using the following equation: corrected total Ca = measured tCa + [(4−albumin) × 0.8].

The last laboratory values minus the baseline results were considered Δ variation.

### Electrical bioimpedance analysis

We used the bioimpedance device InBody S10 tetrapolar segmental bioimpedance device (Biospace Co., Ltd., Korea), performed at the same time of patient inclusion (baseline) and after 6 months of the study (follow-up). The amount of fluid in the lower limbs were evaluated through an electric current between electrodes placed on the ankle and on the thumb and index finger bilaterally, while the patient was lying on supine position. This technique has been previously validated (accuracy of 0.5% and repeatability of 0.3%) for body fluid measurements^16^.

The parameters evaluated by bioimpedance were total body water (TBW), extracellular water (ECW), intracellular water (ICW), and the ECW/TBW ratio. ECW/TBW indicates hypervolemia when values are greater than or equal to 0.40.

### Magnetic resonance imaging acquisition and analysis

Cardiac MRI was performed on 1.5 Tesla scanners (Achieva, Philips Healthcare, Netherlands). Images were obtained during end-expiration breath holds in the supine position, with multiplanar acquisitions in fast Gradient-ECHO steady-state acquisitions with electrocardiographic synchronization, without venous contrast administration. A cardiac MRI expert radiologist (W.I.), who was blinded to the study groups, reviewed all MRI studies, and completed the image postprocessing using commercial software (CVi42 workstation, Circle Cardiovascular Imaging, Canada).

The results were expressed as heart chamber dimensions (end-systole and end-diastole diameters and volumes), right and left ventricular ejection fraction, and left ventricular myocardial mass (absolute values and indexed for body surface area).

Left ventricular hypertrophy was defined according to previously described^17^:

- Women, regardless of age, when LVMI was ≥ 55 g/m^2^
- Men, when the LVMI was ≥ 72 g/m^2^ for those < 64 years old. LVMI was ≥ 70 g/m^2^ for those ≥ 65 years old.

The delta LVMI (ΔLVMI) was defined as the follow-up LVMI minus baseline LVMI.

### Statistical analysis

The results are presented as mean and standard deviation or median and 25/75 percentile according to data distribution, tested using the D'Agostino Pearson test. The comparison between glucose and icodextrin groups was made using chi-square or Fisher when appropriate, for categorical variables and using t-test or Mann–Whitney test for continuous variables. Comparison from baseline to 6-month follow-up was performed using a paired t-test or Wilcoxon in each group. Correlation between two independent variables was assessed by simple regression. Multiple stepwise regression analysis was performed with ΔLVMI as dependent variables and selected variables from univariate analysis. We used GraphPad Software, La Jolla, CA, United States) for graphics and SPSS™ version 22 (SPSS Inc., Chicago, IL, United States) for statistical analysis. The rejection level was set at 5%, that is, p < 0.05.

### Ethical approval

Ethical approval for this study was obtained from Comissão de ética para análise de projetos de pesquisa – CAPpesq do Hospital das Clínicas and from Comissão de Ética em Pesquisa da Universidade Federal de São Paulo—UNIFESP (ID #57602516.6.3001.5505).

## Results

### Baseline

**Figure 1:**
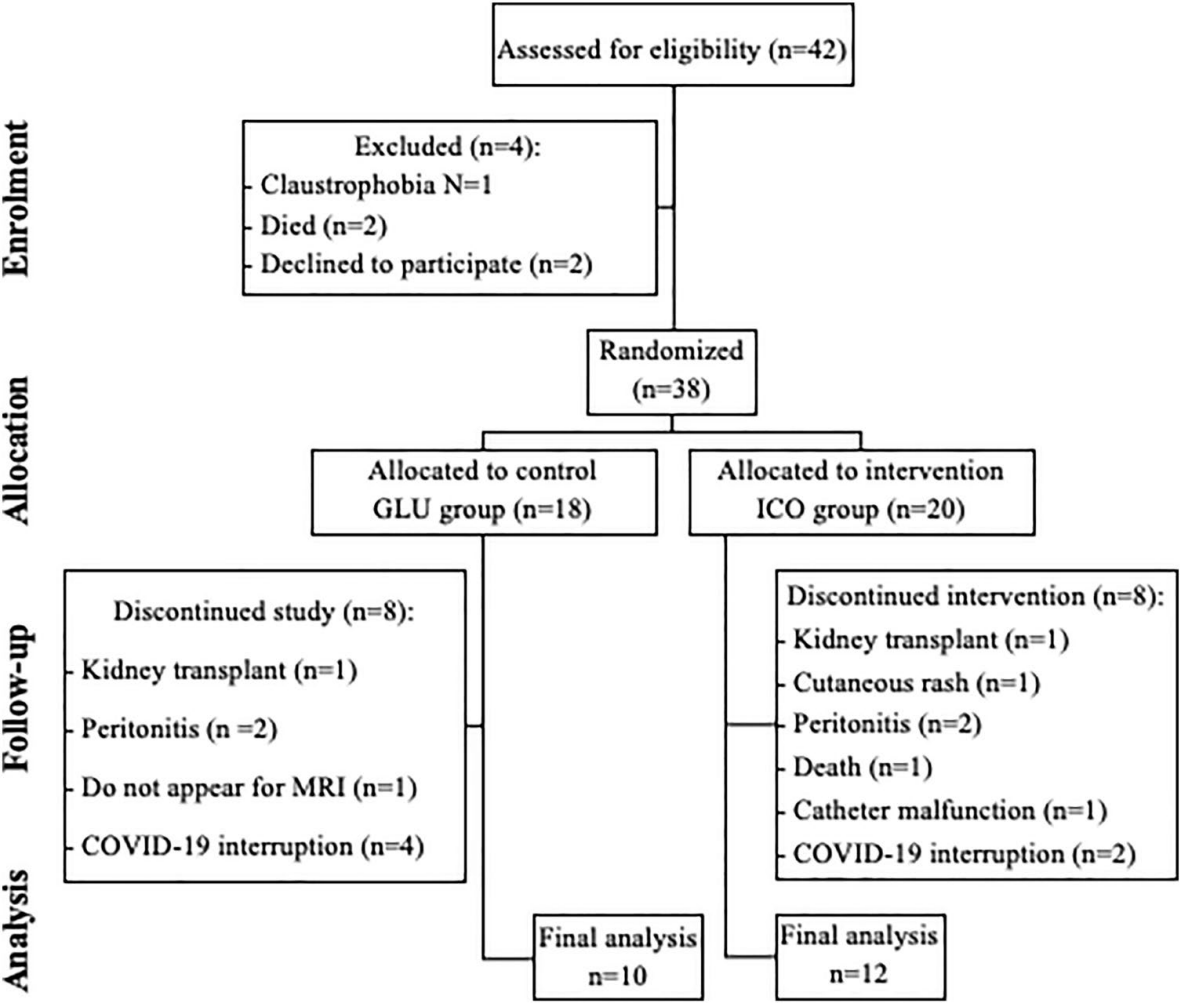
Flowchart of patient selection, follow-up status and data analysis.

A total of 42 patients on PD were screened, 38 were randomized and 22 were included in the final analysis (12 to the GLU group and 10 to the ICO group), as summarized in the CONSORT Flow Chart (Fig. 1).

Table 1 shows characteristics of patients at baseline and follow-up according to the allocated group. Underlying causes of renal failure in GLU and ICO groups were respectively: diabetes (20% vs. 25%), glomerulonephritis (30% vs. 25%), nephrosclerosis (30% vs. 17%), and other causes (20% vs. 33.3%), with no significant difference between groups. Patients were on PD for a median period of 11 (6, 28) months [20 (8, 46) vs. 8 (4, 23) months in GLU and ICO groups, respectively, p = 0.180]. Half of the patients in the GLU group and 25% in the ICO group were on CAPD. The remaining patients were on CCPD. Peritoneal equilibration test revealed that slow, medium, and fast transport status was found in 11%, 78%, and 11% in the GLU group, while 38%, 50%, and 12% in the ICO group, respectively (p = 0.414).

**Table 1:** Clinical and laboratory data at baseline and follow-up according to each group.

	Glucose group N = 10			Icodextrin group N = 12		
	Baseline	Follow-up	Change	Baseline	Follow-up	Change
Age, years	51 ± 18			46 ± 18		
Female sex, %	90			67		
Weight, kg	68.0 ± 15.6	69.3 ± 13.3	0.8 (− 3.1/5.3)	65.5 ± 9.2	66.2 ± 10.0	1.6 (-4.1/4.6)
ECW/TBW	0.46 ± 0.05	0.46 ± 0.05	0 (− 0.01/0.01)	0.39 ± 0.02^#^	0.40 ± 0.12^#^	0 (− 0.01/0.02)
Diuresis, ml	300 (37/925)	100 (0/835)	− 25 (− 262/185)	550 (75/1325)	300 (0/500)*	− 300 (− 575/− 25)
SBP, mmHg	144 ± 26	121 ± 26	− 28 (− 41/10)	140 ± 30	146 ± 19^#^	10 (− 17/17)^#^
DBP, mmHg	87 ± 18	75 ± 16	− 12.5 (− 30.0/− 0.25)	87 ± 19	86 ± 18	− 5.0 (− 17.5/7.5)
UF, ml	775 (575/1.100)	925 (800/1.050)	50 (− 25/250)	880 (625/1050)	1,000 (800/1675)*	250 (− 45/550)
BNP, ng/ml	4.9 (3.5/16.2)	3.9 (2.1/15.3)	1.2 (− 3.0/4.3)	4.9 (1.2/45.0)	17.7 (5.9/42.5)	4.0 (36.6/33.9)
Renal Kt/V	0.68 ± 0.94	0.34 ± 0.59	− 0.05 (− 0.61/0)	0.47 ± 0.51	0.16 ± 0.21	− 0.30 (− 0.35/0)
Total Kt/V	2.38 ± 0.81	2.19 ± 0.44	− 0.04 (− 0.74/0.33)	2.29 ± 1.02	1.71 ± 0.56	− 0.60 (− 1.68/0.46)
Creatinine, mg/dl	8.5 ± 3.4	9.0 ± 3.6	0.1 (− 1.0/1.9)	12.4 ± 4.6^#^	10.8 ± 3.2	− 2.0 (− 4.3/1.1)^#^
CRP, mg/dl	22 ± 26	5 ± 5	− 1.0 (− 55.6/4.7)	8 ± 9	13 ± 16	4.6 (− 0.5/16.0)^#^
Ferritin, ng/ml	297 ± 184	319 ± 282	− 10 (− 82/143)	221 ± 170	211 ± 184	− 29 (− 127/27)
Albumin, g/dl	3.6 ± 0.4	4.0 ± 0.5	0.3 (− 1.0/0.6)	3.9 ± 0.4	3.5 ± 0.7	− 0.3 (− 0.6/0.2)^#^
Cholesterol, mg/dl	202 ± 66	198 ± 70	− 8.5 (− 47.5/52.5)	178 ± 43	156 ± 34	− 16.5 (− 32.5/5.0)
Triglycerides, mg/dl	188 ± 109	193 ± 106	− 5.5 (− 27.2/33.7)	156 ± 174	115 ± 73	− 11 (− 28. 24)
Glucose, g/dl	110.0 ± 26.9	100.5 ± 27.5	− 9.0 (− 20.8/− 3.2)	100.2 ± 55.6	102.7 ± 37.0	0 (− 18/30)
Glycated hemoglobin, %	5.6 ± 0.6	5.2 ± 0.7	− 0.2 (− 0.3/0.2)	5.7 ± 1.2	5.9 ± 1.4*	− 0.3 (1.4/0.6)
Hemoglobin, g/dl	11.2 ± 1.9	11.2 ± 1.9	− 0.2 (− 0.5/1.0)	11.3 ± 2.3	10.9 ± 1.4	0 (− 2.3./0.6)
Hematocrit, %	34.3 ± 5.8	33.5 ± 5.8	− 1.2 (− 2.4/2.5)	34.9 ± 7.3	32.9 ± 4.4	− 2.2 (− 6.3/1.1)
iCa, mg/dl	4.79 ± 0.19	4.84 ± 0.23	0.07 (− 0.20/0.07)	4.78 ± 0.42	4.79 ± 0.39	0.05 (− 0.21/0.18)
tCa, mg/dl	9.8 ± 0.7	10.0 ± 1.0	0.48 (− 0.52/1.03)	8.9 ± 0.6#	8.7 ± 0.7#	0.3 ( − 0.3/0.9)
25(OH)Vit.D, ng/ml	19.8 ± 6.4	23.1 ± 8.9	2.6 (− 4.0/8.9)	19.7 ± 6.2	26.2 ± 10.7*	4.9 (0.05/14.2)
PTH, pg/ml	293 (176/475)	230 (129/697)	− 38 (− 246/67)	392 (245/1032)	327 (228/559)	25 (− 49/234)
FGF- 23, pg/ml	343 (104/3,834)	189 (45/4,631)	0 (− 68/153)	1,032 (175/4,761)	1,421 (248/4,738)	146 (0/3,446)
AP, UI/ml	76 (67/104)	76 (54/120)	1.5 (− 9.0/11.2)	97 (73/122)	94 (59/113)	4.0 (− 8.8/28.5)

ECW/TBW, extracellular water/total body water; SBP, systolic blood pressure; DBP, diastolic blood pressure; UF, ultrafiltration; BNP, brain natriuretic peptide; CRP, C-reactive protein; iCa, ionized calcium; tCa, total calcium corrected by serum albumin; 25(OH)Vit.D. 25 hydroxy-vitamin D; PTH, parathyroid hormone. Values are expressed as mean ± SD or median (25/75).

*p < 0.05 vs. baseline in each group; #p < 0.05 vs. glucose group.

**Table 2:** Magnetic resonance imaging at baseline and follow-up according to each group.

	Glucose group N = 10			Icodextrin group N = 12		
	Baseline	Follow-up	Change	Baseline	Follow-up	Change
LV ejection fraction, %	64.2 ± 10.8	62.1 ± 15.4	3.7 (− 5.1, 9.1)	54.0 ± 5.9^#^	52.1 ± 10.9^#^	− 2.9 (− 6.5, 5.8)
LA stroke volume, ml/m^2^	43.8 ± 17.2	40.5 ± 13.7	− 3.0 (− 18.5, 11.1)	55.1 ± 24.2	52.4 ± 19.3	− 3.7 (− 24.5, 22.0)
RV end- diastolic dimension, ml/m^2^	66.3 ± 20.8	68.4 ± 18.9	1.4 (− 8.8, 10.0)	83.7 ± 20.0	83.1 ± 17.8	− 0.2 (− 13.0, 14.9)
RV end-systolic dimension, ml/m^2^	23.5 ± 9.1	24.6 ± 10.0	0.3 (− 3.8, 4.3)	29.5 ± 9.4	36.5 ± 17.3	4.7 (− 6.5, 24.2)
LV end-diastolic dimension, ml/m^2^	69.8 ± 17.0	79.6 ± 23.0	6.1 (− 11.0, 11.1)	98.9 ± 37.4^#^	100.4 ± 27.7	0.4 (− 23.4, 21.8)
LV end-systolic dimension, ml/m^2^	25.8 ± 12.3	32.6 ± 24.8	0.5 (− 6.5, 4.9)	46.7 ± 22.6^#^	50.3 ± 20.7	3.7 (− 16.0, 18.0)
LVMI, g/m^2^	66.2 ± 13.5	65.7 ± 18.3	− 3.9 (− 10.7, 2.2)	89.1 ± 28.9^#^	88.1 ± 28.9^#^	5.2 (− 26.8, 16.8)
Inferolateral wall thickness, mm	7.7 ± 1.6	8.3 ± 1.6	1.0 (− 1,0, 2.0)	8.3 ± 1.8	8.6 ± 3.6	0 (− 1.7, 2.5)
Anteroseptal wall thickness, mm	9.8 ± 2.2	10.0 ± 2.6	0 (− 1.5, 1.5)	11.1 ± 2.2	11.2 ± 3.0	− 0.5 (− 1.7, 1.7)

LV, left ventricular; LA, left atrium; RV, right ventricular; LVMI, left ventricular mass index. Values are expressed as mean ± SD or median (25/75).

^#^p < 0.05 vs. glucose group.

According to ECW/TBW ratio, 4 (36.8%) and 8 (80%) of patients from GLU and ICO groups, respectively, were considered hypervolemic (p = 0.044). BNP was above the superior limit in all included patients, with no difference between groups.

The number of antihypertensive drugs was also similar between GLU and ICO groups [1.5 (0.8, 2.2) vs. 2.0 (1.0, 3.7), respectively, p = 0.456). ACE/ARB has been prescribed to 60% and 75% of patients from GLU and ICO groups, respectively (p = 0.452). ECW/TBW was lower in the ICO group at baseline, as well as iCa. As shown in Table 2, patients allocated for the ICO group had higher LVMI, LV end-diastolic dimension, LV end-systolic dimension, and lower ejection fraction. Seven patients in GLU group and 10 in the ICO group met MRI criteria for LVH (p = 0.748). LVMI correlated with creatinine (r = 0.823, p = 0.006), diastolic blood pressure (r = 0.832, p = 0.005), and hematocrit (r = -0.495, p = 0.022).

### Follow-up

Recovery from LVH was observed in only 2 patients who were randomized to the GLU group. LVMI decreased in 5 patients from each group and remain stable or increased in the remaining patients, which was similar in both groups (p = 0.528).

There was an increase in ultrafiltration volume and a decrease in residual diuresis in the ICO group (Table 1). No other change was observed from baseline to follow-up. Changes in systolic blood pressure (better control in the GLU group), creatinine (higher drop in the ICO group), and C-reactive protein (decreased in the GLU group) significantly differed between groups. BNP decreased in 28.6% and 42.9% of patients from GLU and ICO groups, respectively, a non-significant difference (p = 0.577).

**Figure 2:**
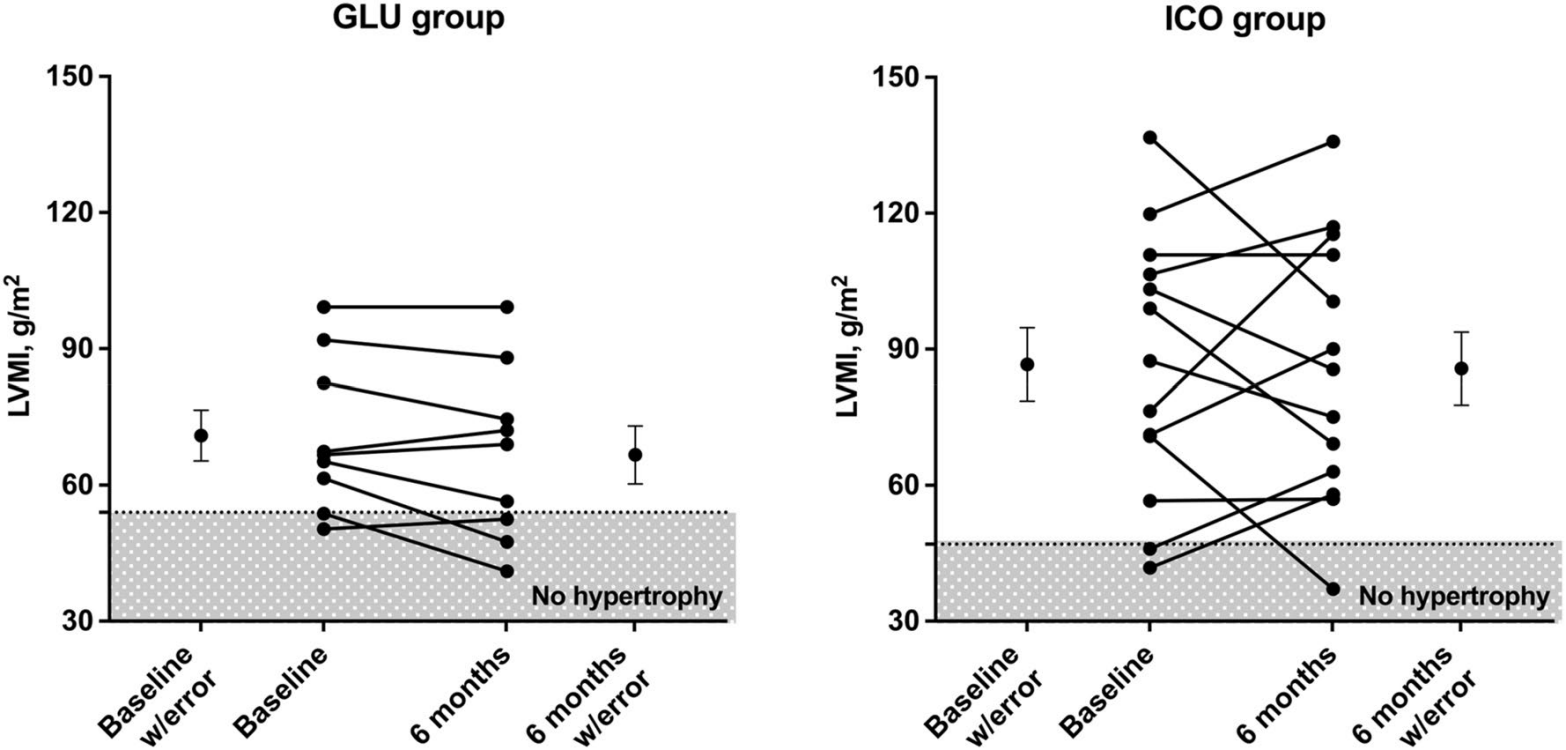
Changes in left ventricular mass index (LVMI) from baseline to 6 months in each group. Dashed lines represent limit of normality in each group. Patients in the shade area had no left.

As shown in Table 2, no change was verified in MRI parameters in GLU and ICO groups during follow-up, At the end of follow-up, patients from the ICO group persisted with lower ejection fraction and higher LVMI than patients in the GLU group. Figure 2 illustrated the individual changes of LVMI in each group. Both groups had a decrease in LVMI. ΔLVMI correlated with ΔBNP (r = 0.566, p = 0.044), and had a tendency toward ΔiCa (r = − 0.512, p = 0.051). ΔLVMI was similar according to sex, presence of diabetes, number of antihypertensive drugs, modality of dialysis (CCPD vs. APD), use of diuretics, ACE/ARB, calcitriol, cinacalcet, phosphate binder, cholecalciferol (p > 0.05 for all comparisons). Multiple linear regression in a model adjusted for ΔiCa, baseline LVMI and study group revealed that ΔLVMI was dependent on ΔBNP (partial correlation 0.670, p = 0.048), explaining 37.1% of its variability.

## Discussion

This clinical trial compared cardiac MRI changes in patients on PD treated with icodextrin-based dialysate with those receiving glucose-based dialysate. Although there was an increase in ultrafiltration volume with icodextrin solution, patients from this group remained with a higher fluid overload and higher systolic blood pressure levels. Changes in LVMI were dependent on BNP changes, which did not differ between the two groups.

There is an international variation in the icodextrin use, whereas 50% of patients from Australia/New Zealand, Canada, Japan, and the United Kington have this prescription, while this solution is hardly used in Thailand^18^ or Brazil. Icodextrin was developed to provide sustained ultrafiltration over an extended dwell. As expected, patients in the ICO group showed an increase in ultrafiltration volume. Icodextrin was well tolerated. Only one adverse event, i.e. an episode of skin rash was observed, a complication previously described in the literature^19^. Another aspect observed in the ICO group was the improvement of the lipide profile. Patients experienced a no statistically significant reduction in cholesterol and triglycerides levels. Glycated hemoglobin was within recommended target ranges in all patients, so an additional reduction was not expected. Metabolic improvement has been extensively reported in the literature in association with the use of icodextrin^12,20–22^, and it was beyond the scope of the current study.

We used bioimpedance to establish the volume status, considering the ECW/TBW ≥ 0.4 as a measure of fluid overload. The use of bioimpedance has been considered a reliable tool to assess fluid status in patients on PD^23^. In addition, hypervolemia has been associated with a higher LVM^24^. This finding suggests that the control of fluid overload should be a target to reduce LVH in patients on PD. Indeed, the changes in BNP was independently associated with ΔLVMI, corroborating to this hypothesis of the impact of fluid overload as a predictor of LVH. LVH is a high prevalent risk factor for mortality in patients on PD^25^. We observed that 77.3% of included patients presented LVH at study entry, which was similar to previous reported findings^26,27^.

Also, our results showed that ΔLVMI was dependent on the ΔBNP, suggesting that volume control has a role in the LVH. There was no clinical difference between groups regarding clinical signs and symptoms of hypervolemia. The clinical assessment, however, of fluid status is relatively difficult in patients on dialysis. In this regard, tools such as the diameter of inferior vena cava assesses by transthoracic echocardiography, BNP, N-terminal pro-brain, and bioimpedance spectroscopy have been described. The N-terminal BNP has been used as a marker of volume overload in patients on PD. N-terminal BNP correlates with survival^28^ and fluid status assessed by bioimpedance^29^. Kidney function and myocardial stretch are the main determinants of NT-proBNP and BNP levels, which may increase 20-fold the upper limit of assays^30^, in agreement with our findings. In a recent systematic review and meta-analysis, both NT-proBNP and BNP levels were associated with clinical outcomes^31^ in patients with end-stage renal disease.

Like our findings, a previous study using MRI in a subgroup of 82 patients^12^, did not observe an improvement in LVM after 6 months of intervention (from 128 ± 37 to 132 ± 60 g in the GLU group *vs.* 128 ± 45 to 124 ± 39 g in the ICO group, p = 0.83). However, the comparison of both studies is difficult because the mentioned study was a sub-analysis and did not adjust LVM for the body surface area.

We failed to demonstrate that icodextrin, by increasing UF volume, would reduce left ventricular mass. Several reasons might have contributed to this result: (1) the increase in UF has not been accompanied by a reduction in fluid status (ECW/TBW increased in the ICO group). Physicians possibly attempted to continue reducing peritoneal glucose load in patients receiving icodextrin. Therefore, once adequate ultrafiltration was not an issue in this group, progressively volume overload could occur; (2) salt and water intake were not rigidly controlled, and we have no data on salt elimination. Salt elimination can vary depending on the PD modality and 50% of patients in each group was on CAPD; (3) most of patients had low or medium peritoneal transport characteristics, and these patients usually do not have difficulties increasing UF using a glucose-based solution. However, it should be mentioned that we have no data on glucose load in each group; and 4. blood pressure levels, a well-recognized risk factor for LVH was higher in the icodextrin group. In a recent observational study, Tangwonglert T^26^ showed that LVMI assessed by echocardiography was not associated with 24-h PD ultrafiltration. The authors also showed that volume expansion was more significant than blood pressure levels in determining LVH; (4) finally, patients from the glucose group have lower LVMI and lower ejection fractions than patients from the icodextrin group at the randomization. This difference might have impacted the results since there was not much room for improvement in the glucose group, regardless of any chosen approach.

In summary, icodextrin should be part of therapeutic options, mainly for those patients who do not respond to other adjustments in PD prescription to improve volume status, as suggested by a recent meta-analysis^32^. However, icodextrin may not be capable of improving LVMI in patients on PD, on its own.

Our study has important strengths, including the prospective and randomized design, the use of bioimpedance analysis to assess fluid overload, and the application of cardiac MRI to assess LVH. Some limitations are important to be mentioned when interpreting these results. First, the inclusion of relatively young patients with a low prevalence of diabetes precludes the generalizability of data. Second, this study was hampered by the COVID-19 pandemic, which limited and reduced the study sample size. Third, the follow-up of 6 months might have not be long enough to appreciate changes in LVM^8,12^. Finally, we did not control diet aspects. 

In conclusion, the use of icodextrin-based solution in prevalent patients on CAPD or CCPD compared with glucose-based solution was not able to improve LMV, a result that should be interpreted considering baseline differences between groups. A larger randomized trial with longer follow-up period may be needed to show changes in LVM in this patient population, because it is a highly prevalent disorder that progresses over time in these patients.

## Data Availability

Data is publicly available at OSFHome: https://osf.io/hb2wm/.
